# Supplementary material for: Tailoring baker’s yeast Saccharomyces cerevisiae for functional testing of channelrhodopsin
Source: PLoS One. 2023 Apr 13;18(4):e0280711. doi: 10.1371/journal.pone.0280711 (PMC10101416; doi:10.1371/journal.pone.0280711)
Supplement: S1 Fig — (A) Fluorescence images of SHY4 cells transiently expressing GFP tagged K+ channel KcvPBCV1 (KcvPBCV1::GFP). Scale bar = 5 μm. (B) Serial dilutions of K+ uptake deficient SHY4 cells transformed with KcvPBCV1::GFP plasmid or the corresponding empty vector (ev). Cells spotted on SD-ura plates with either high (100 mM KCl, top) or without K+ supplementation and incubated for 72h. KcvPBCV1 rescues growth of SHY4 cells on SD-ura medium without additional K+ (bottom row). For experimental details see [1]. [1] Gebhardt M, Hoffgaard F, Hamacher K, Kast SM, Moroni A, Thiel G. Membrane anchoring and interaction between transmembrane domains is crucial for K+ channel function. J. Biol. Chem. 2011; 286:11299–11306. (PDF) [file pone.0280711.s001.pdf]

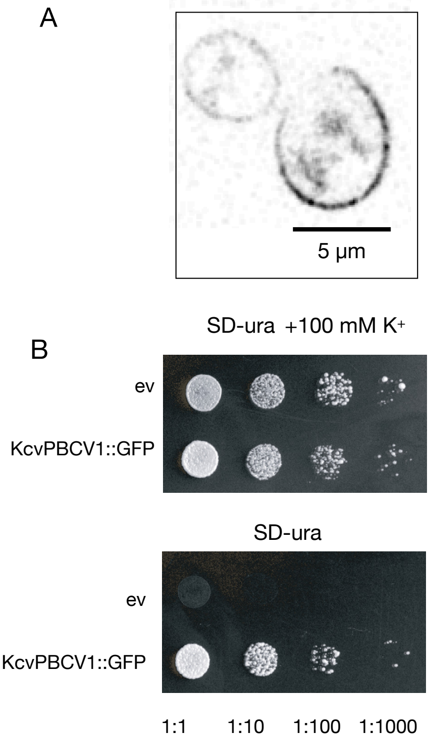

Figure S1. Functional complementation of SHY4 cells by K^+ channel. (A) Fluorescence images of SHY4 cells transiently expressing GFP tagged K^+ channel KcvPBCV1 (KcvPBCV1::GFP). Scale bar = 5 µm. **(B)** Serial dilutions of K^+ uptake deficient SHY4 cells transformed with KcvPBCV1::GFP plasmid or the corresponding empty vector (ev). Cells spotted on SD-ura plates with either high (100 mM KCl, top) or without K^+ supplementation and incubated for 72h. KcvPBCV1 rescues growth of SHY4 cells on SD-ura medium without additional K^+ (bottom row). For experimental details see [1]

[1] Gebhardt M, Hoffgaard F, Hamacher K, Kast SM, Moroni A, Thiel G. Membrane anchoring and interaction between transmembrane domains is crucial for K^+ channel function. J. Biol. Chem. 2011; 286:11299-11306.
