# Supplementary material for: Tailoring baker’s yeast Saccharomyces cerevisiae for functional testing of channelrhodopsin
Source: PLoS One. 2023 Apr 13;18(4):e0280711. doi: 10.1371/journal.pone.0280711 (PMC10101416; doi:10.1371/journal.pone.0280711)
Supplement: S2 Fig — Serial dilutions of all-trans retinal producing K+ uptake deficient SHY4 cells transformed with either a plasmid for constitutive expression of N/K-ChR2-eYFP or the corresponding empty vector (ev). Cells spotted on SD-ura plates with either high (100 mM KCl, top) or a without K+ supplementation and incubated for 72h. Colonies grow only on SD-ura medium with high K+ concentration independently of incubation in the dark (left) or in blue light (right). (PDF) [file pone.0280711.s002.pdf]

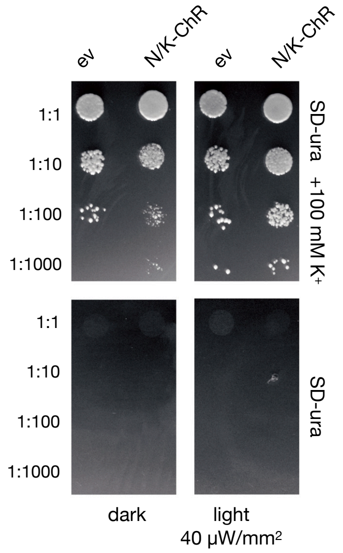

Fig. S2. N/K-ChR2-eYFP expression does not restore growth of SHY4 cells on low K^+ agar medium. Serial dilutions of all-*trans* retinal producing K^+ uptake deficient SHY4 cells transformed with either a plasmid for constitutive expression of N/K-ChR2-eYFP or the corresponding empty vector (ev). Cells spotted on SD-ura plates with either high (100 mM KCl, top) or a without K^+ supplementation and incubated for 72h. Colonies grow only on SD-ura medium with high K^+ concentration independently of incubation in the dark (left) or in blue light (right).
