# Supplementary material for: Tailoring baker’s yeast Saccharomyces cerevisiae for functional testing of channelrhodopsin
Source: PLoS One. 2023 Apr 13;18(4):e0280711. doi: 10.1371/journal.pone.0280711 (PMC10101416; doi:10.1371/journal.pone.0280711)
Supplement: Attachment — Submitted filename: Dear reviewer.docx [file pone.0280711.s005.docx]

Dear reviewer, thanks for pointing out issues that might have been not clear yet in the previous version of the manuscript. We have now amended the manuscript accordingly:

**Reviewer:**

I understand the author response to my original comment (2). However, I am still unconvinced with the author explanation mostly because I am unable to confidently assess the localizations of N/K-ChR2-eYFP with the microscopy images shown in Fig. 5A. For most the cells, the protein seems to be localized internally but it can’t be determined in which organelle it is localized. Ideally, they should show the co-localization of N/K-ChR2-eYFP with organelle markers. Again, I have the impression that N/K-ChR2-eYFP is mostly internalized with a small proportion localized at the PM.

**Our response:**

We do not question fluorescence in internal membranes, but GFP tagged channel protein is for sure visible in the plasma membrane of **all cells,** which express the channel protein. This occurs on a variable background ranging from no fluorescence signal inside the cell (cell now indicated in Fig. 5) to an intense labeling of internal structures. By adding two additional paragraphs we want to make the important point that already the presence of GFP fluorescence in the plasma membrane is sufficient for using the yeast system for complementation assays. This is irrespective of the presence/absence of cytosolic signals.

To strengthen our message, we have:

a. added a quantitative analysis on fluorescence localization in the plasma membrane versus the cell interior. This analysis confirms the general presence of GFP fluorescence in the plasma membrane not only in a few images but in a large cohort of analyzed cells (page 5).
b. We have added an experiment (page 3, Fig. S1) in which we confirm, with the help of a positive control, that a weak GFP signal from a channel protein in the plasma membrane is sufficient for using the K^+^ uptake deficient yeast strain for complementation assays. Such a complementation can only be interpreted in the context of channel activity in the plasma membrane.
c. We have not performed colocalization assays since for our application it is not relevant to identify the cytosolic localization of the protein. From the biogenesis of a membrane proteins, we know that we will find positive colocalization with the Golgi and the ER. Although this information is interesting in the context of channel synthesis and sorting it will not provide crucial insights into the positive or negative outcome of our complementation assays.

**Reviewer:**

Please indicate how many biological replicates for experiments in figure legends. The authors responded that “We are surprised by this comment because the relevant numbers are already given in the figure legends where relevant, i.e. when statistical information is provided (Figs. 2, 4, 5 and 7)”. I am sorry for the confusions, but I mean that it is not indicated in the method nor in figure legends if the other experiments were repeated at least in triplicates such as Fig. 1A, 1B, 1C, 1D, 2B (three values means three independent biological replicates?), 3A, 3B, 3C, 4C, 4D, 5A, and 6. Alternatively, the authors can state in the method section that all experiments have been conduced at least for three independent biological replicates if it is the case.

**Our response:**

We included an extra chapter on “statistics” in the material and method section in which we state all the information on experimental replicates etc.
